# Thickness Thresholds for Generating Plasmonic Surfaces via Solid-State Dewetting of Ultrathin Silver Films on Soft Polymeric Substrates

**DOI:** 10.1021/acsomega.6c00698

**Published:** 2026-06-17

**Authors:** Jakob Nüßlein, Sagnik Mondal, Steffen Strehle

**Affiliations:** Institute of Micro- und Nanotechnologies, Microsystems Technology Group, Technische Universität Ilmenau, Ilmenau 98693, Germany

## Abstract

This study demonstrates a dewetting process on polymeric polydimethylsiloxane (PDMS) substrates, enabling the fabrication of soft plasmonic masks based on silver thin films of varying thicknesses, applied to both planar and structured surfaces. The adaptation and suitability of thermal dewetting processes, typically employed on rigid substrates, for the creation of plasmonic applications are investigated. A distinct threshold behavior was observed: ultrathin films with thicknesses between 1 and 9 nm exhibited pronounced plasmonic resonance, whereas thicker layers of 13–19 nm showed no measurable response. These results indicate that both the initial thin-film thickness and the resulting nanoparticle geometry critically influence the collective plasmonic properties. Thinner layers produced higher *Q* factors and sharper resonance dips, with the maximum *Q* factor of 4.58 observed at an initial film thickness of 3 nm, indicating that this thickness is most suitable for plasmonic sensing. In contrast, an initial thickness of 9 nm produced the strongest overall plasmonic response, making it preferable for applications where resonance strength is the primary factor, such as plasmonic lithography or surface-enhanced Raman spectroscopy. The long-term stability of the fabricated samples was additionally examined over six months, revealing that the plasmonic response remains detectable, albeit with a significant decrease in intensity. Structural and optical analyses confirm the process’s compatibility with structured substrates, enabling precise nanostructuring while preserving underlying surface features. The simplicity of the proposed method makes it a practical alternative to the typically more complex techniques used in plasmonic system fabrication.

## Graphical abstract

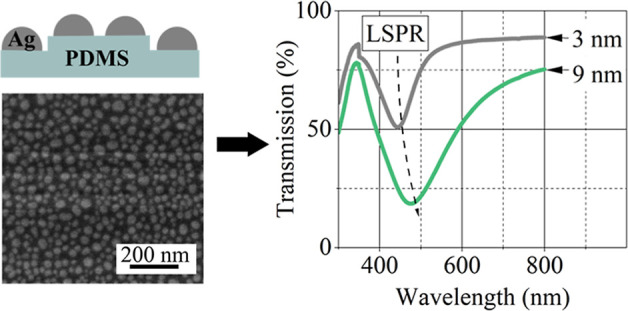

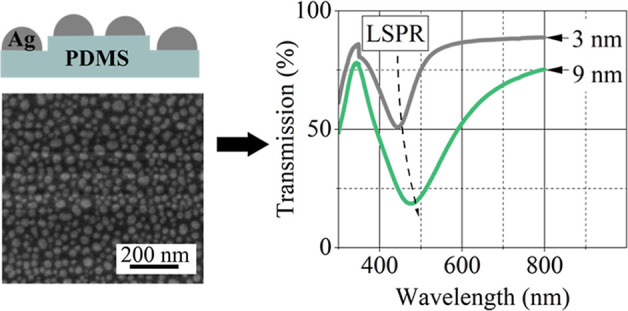

## 1 Introduction

The plasmonic effect, resulting from the collective oscillations of conduction electrons at metal–dielectric interfaces, allows light to be confined to extremely small regions. This distinctive property is especially advantageous for applications demanding ultrahigh resolution, including the optical lithography fabrication of nanoscale devices and sensors. Plasmonic lithography, for instance, is a type of subwavelength lithography that exploits the optical properties of metal nanostructures. In this context, the so-called plasmonic masks are an essential part in the process, which enable patterning beyond the diffraction limit of conventional optical lithography. This capability has been demonstrated already under experimental conditions.^1,2^ In addition to lithographic applications, the strongly localized field enhancement of plasmonics is also utilized in other fields like in energy devices (e.g., solar cells),^3,4^ spectroscopy methods such as surface-enhanced Raman spectroscopy (SERS),^5^ catalytic processes,^6^ and various sensing technologies^7^ including refractive index sensing^8^ or the detection of different biological or chemical compounds.^9^

However, achieving reliable and scalable plasmonic performance across these diverse applications remains a persistent challenge. In particular, the implementation of well-controlled plasmonic nanostructures on flexible or large-area substrates, which are critical for next-generation sensing and lithography, has been investigated with different approaches^6,10–12^ but has not yet been fully explored.

Each application area imposes specific requirements, particularly with regard to the plasmonic masks used. Key challenges include the limited effective field enhancement area, positioning accuracy, material constraints, and manufacturing complexity, especially for large working areas, which together hinder widespread use and industrial scalability. Current research efforts focus on overcoming issues such as low exposure depth and insufficient pattern contrast^13,14^ as well as developing high-precision positioning tools capable of subnanometer accuracy for improved control.^15^

In addition to the challenges emerging from the application requirements, the material choice of the plasmonic masks is also critical for achieving optimal plasmonic performance in the intended wavelength region. Metals like gold are commonly used due to their excellent plasmonic and noble properties,^16,17^ but limitations in the tunability of their plasmonic resonance have sparked interest in alternative materials such as silver and aluminum, which are particularly promising for ultraviolet (UV) and near-UV applications.^18–20^ Further materials, such as magnesium and indium, also theoretically exhibit promising properties in this wavelength region, but their application is often limited by manufacturing challenges or low chemical stability.^18^

Conventional methods to create metallic nanostructures for plasmonic masks, such as focused ion beam and electron-beam lithography, provide exceptional precision and resolution while offering flexibility and freedom in pattern design but are limited by their low throughput and small patterning areas as well as their challenging compatibility with large or flexible substrates. They are also sensitive to substrate properties like surface roughness and tilt,^2,21,22^ with proximity effects posing an additional challenge in accuracy for electron beam lithography.^23^

In contrast, scalable fabrication strategies like colloidal lithography,^24^ drop-casting,^25^ and solid-state dewetting^26,27^ have emerged as cost-effective alternatives. These methods can, in principle, produce large-area plasmonic structures but are still underexplored for mask fabrication, especially on soft substrates. They must be carefully designed to ensure controllable outcomes, as inappropriate parameter settings may result in uneven size distributions or random spatial arrangements of the fabricated nanostructures.^24,28^ Among these, solid-state dewetting represents a straightforward and promising approach. Various adaptations of this process have demonstrated reproducible results on substrates such as silicon or glass, but their adaptation and application to soft or polymeric substrates remain largely unexplored. Notable modifications include dual thermal dewetting,^29^ templated dewetting,^30^ bilayer dewetting,^31,32^ or laser-induced dewetting.^33,34^ These adaptations have been widely studied in the literature and have shown to improve control over size, spacing, and arrangement of nanostructures, making them particularly suitable for applications like plasmonic masks where uniformity and reproducibility are essential. Compared to traditional methods, these approaches are specifically advantageous because of their ability and suitability to be scaled down or up depending on the application needs, with only small adaptations in the process parameters.^32^ However, the application of these dewetting-based approaches to soft or polymeric substrates remains largely uninvestigated,^35^ despite their clear potential for flexible plasmonic systems.

This research gap motivates the present study. We aim to address some of the aforementioned challenges and to explore a scalable, large-area fabrication technique for plasmonic masks applicable in sensing and lithography applications. Our work introduces particularly a hybrid plasmonic stamp comprising a polydimethylsiloxane (PDMS) polymer layer and a nanostructured silver layer fabricated via thermal dewetting. We adapt a solid-state dewetting originally designed for rigid standard substrates (e.g., Si, glass) to enable the development of soft plasmonic masks. Various temperatures were evaluated in this regard. Convincing results were achieved by applying a temperature of 200 °C for 1 h, which is consistent with methods employed by refs 36 and 37 Lower temperatures yielded weaker plasmonic resonances of the samples, while higher temperature affected the mechanical and optical properties of the PDMS layer as also shown in ref 38. Furthermore, samples with varying initial silver thicknesses (1 to 19 nm) are compared and analyzed in our study, where we found distinct plasmonic thickness thresholds. We demonstrate the feasibility and flexibility of the method by using flat and structured substrates. The results demonstrate a scalable and reproducible route for fabricating soft plasmonic masks that combine mechanical flexibility with strong, localized field enhancement, compatible with standard UV lithography workflows. This approach bridges the current gap between high-precision yet low-throughput nanolithography methods and scalable, flexible plasmonic fabrication techniques, thereby expanding the practical applicability of plasmonic stamps in sensing and subwavelength patterning.

## 2 Materials and Methods

This section describes the materials used, analytical methods applied, and fabrication tools employed.

### 2.1 Materials and Fabrication Tools

For the master, we used unstructured, planar glass substrates and silicon substrates with grid structures. The glass substrates were prepared from commercially available optical microscopy slides (2305501206, DWK Life Sciences), and silicon substrates featuring a grid structure with a pitch of 40 μm, a width of the structures of 30 μm, and a height of 4.2 μm were fabricated using standard UV lithography, followed by a dry etching process to transfer the structure from the photoresist into the silicon substrate. Square-shaped molds were produced by 3D printing using an acrylonitrile styrene acrylate filament with openings of (2 × 2) cm^2^ and heights of 2 mm to ensure uniform PDMS thickness and dimensions across all samples. To prevent leakage during curing, the molds were fixed to the silicon masters using a double-sided adhesive tape (tesa 05338, tesa SE). For our soft polymeric layer, we used PDMS (Sylgard 184, Dow Corning) that was prepared in a standard process also often used in nanoimprint applications. The base and cross-linker were mixed in a 10:1 ratio and then stirred at 2000 rpm for 5 min. Afterward, four degassing cycles were performed in a desiccator for 8 min each. After pouring the PDMS, it was cured for 4 days at room temperature (22 °C). Metallization was performed using an electron beam evaporator (PVD-Cluster CS400ES, Von Ardenne) equipped with an internal quartz crystal microbalance (QCM) for precise film thickness control. This QCM got calibrated initially by the manufacturer of the device, and its lifetime cycle and resonance got monitored during the operation through the software. For thermal dewetting, samples were placed on a hot plate (Hei-PLATE Mix“n”Heat Core, Heidolph) featuring an internal temperature control loop. After reaching the target temperature, an additional 5 min was allowed for stabilization before sample placement. A temperature of 200 °C under ambient conditions was identified as the optimal value, based on preliminary experiments described in more detail in Section 3.1.

### 2.2 Characterization of the Plasmonic Masks

The fabricated soft masks were characterized by using several complementary techniques. The samples were examined before and after the dewetting step by using a Nikon Eclipse L300D optical microscope equipped with darkfield illumination to verify that no damage to the PDMS occurred during the dewetting process. The structured PDMS samples were additionally analyzed at all process steps using a 3D laser scanning microscope (LSM, LEXT OLS4100, Olympus) to verify that the master structures were accurately transferred into the polymer and that no deformation or bending of the structures was induced by either the metalization or dewetting step. The formation of nanoparticles was further verified by scanning electron microscopy (SEM S-4800, Hitachi). The particle sizes were analyzed using the open-source software ImageJ version 1.54 g, where the particle area was approximated as circular to calculate the mean diameter. To investigate the interparticle distances, nearest-neighbor analysis was performed. The values were obtained using the BioVoxxel toolbox, an ImageJ plugin.^39^

The optical properties of the samples were investigated using a UV–vis-NIR Spectrometer (Cary 5000, Varian) in transmission mode over the wavelength range of 800–300 nm. The instrument is equipped with multiple light sources to cover distinct wavelength ranges. During the light-source transition at 350 nm, a transient disturbance can occur, producing a localized region of increased noise. Before each measurement, the instrument was calibrated with the internal software, and reference spectra of blank PDMS samples were acquired to ensure that transmission changes originated from the structured surfaces. All measurements were performed within 1 week after metallization, with an additional measurement taken after six months to assess the long-term stability of the soft plasmonic masks.

To compare the different samples, an approach similar to that described in refs 40 and 41 was used. Different parameters derived from the transmission measurement were used and processed with OriginPro Version 2023 software (OriginLab Corporation) to extract the baseline and minimum values as well as the minimum wavelength λ_0._ The magnitude was defined as the difference between baseline and minimum transmission. A half value was then calculated, and the corresponding wavelengths on both sides of the resonance were used to determine the full width at half-maximum (FWHM). To compare different samples, a quality factor (*Q* factor) was calculated as *Q* = λ_0_/FWHM, which summarizes the characteristics of a specific sample.

## 3 Results and Discussion

In the following, the results for soft plasmonic masks that were fabricated by using two different types of masters are presented. First, some preliminary considerations and the selected workflow for the samples are introduced, after which the obtained results are discussed. The samples are distinguished according to the employed master: (I) planar PDMS prepared from microscope slides and (II) structured PDMS prepared from an in-house-fabricated silicon master. This differentiation enables a systematic comparison of how substrate structuring influences the film morphology, dewetting behavior, and the resulting optical properties. Additionally, the two most promising samples were reanalyzed after six months to investigate the long-term stability of the fabricated samples.

### 3.1 Preliminary Considerations and Material Selection

PDMS was utilized as the polymeric layer for the soft plasmonic mask due to its mechanical flexibility, chemical stability, and high transparency to UV radiation.^42,43^ Its high UV transparency enables effective use in lithographic and sensing processes, where light must pass through the PDMS layer, serving as a substrate, with minimal energy loss. The chemical robustness of PDMS enables, furthermore, a reliable use in various analytical or chemical sensing applications.^43,44^ Additionally, due to our intended approach involving direct mechanical contact, its mechanical flexibility is essential for use as a soft plasmonic mask. Acting as a spring damper system,^45^ PDMS facilitates correction of potential tilts in the setup while simultaneously reducing the mechanical force applied to the plasmonic nanostructures at the surface, thereby protecting their integrity and preventing potential damage.

Since the goal is to fabricate samples for plasmonic applications in the UV and near-UV range, the most promising materials for the creation of plasmonic resonators are currently metallic layers such as aluminum and silver. In the present experiments, silver was selected as the thin-film material. Aluminum theoretically offers superior plasmonic capabilities in the UV range compared to most other materials,^18^ but it is prone to surface oxidation, which redshifts its plasmonic response.^19^ The resulting oxide layer can both protect against further oxidation and hinder processes such as dewetting due to its strong surface adhesion.^46^ Despite this, solutions exist,^32,47^ but they have only been reliably tested for silicon and glass substrates. Silver is likewise prone to oxidation and corresponding redshifting of the plasmonic resonance. However, silver oxide does not form a protective layer but instead oxidizes completely, which degrades its long-term stability. Despite this, silver possesses tunable plasmonic properties ranging from the visible down to the UV spectrum and is well suited for thermal dewetting on standard substrates.^18,20^

Nevertheless, dewetting on polymer substrates such as PDMS has, to date, received relatively limited attention in the literature. A related study employed a comparable approach but utilized gold as the metallic material instead, with the resulting structures serving as catalysts for a hydrosilylation process.^6^ Standard solid-state dewetting of silver thin films on fused silica or Si often uses high temperatures (300–1000 °C) or rapid thermal annealing tools.^34,48–50^ However, due to the thermal sensitivity of polymers,^38,51^ lower temperatures must be targeted to realize an appropriate dewetting process.

Therefore, we exemplarily tested 3 and 5 nm-thick silver films on PDMS considering parameters from rigid substrates mentioned in previous studies.^36,37,50^ Both investigated thicknesses showed a similar trend that can be seen in the transmission spectra of the 3 nm samples that are exemplary displayed in Figure 1a. The explored temperatures ranged from 100 to 500 °C in 100 °C increments, with an additional step added at 250 °C. This additional step was added because we expected the thermal stability of PDMS to be at least 300 °C and we assumed that higher temperatures would lead to improved optical responses. The annealing duration was always 1 h. A representative macroscopic image of a sample before the dewetting step is shown in Figure 1b. PDMS degradation began at 300 °C, marked by a loss of flexibility. The observed degradation temperature matches with experimental results as reported in ref 52. A complete PDMS failure was observed at 500 °C being characterized by whitening and ash-like residues as shown in Figure 1c,d. Using the lowest temperature of 100 °C worked but yielded a slightly weaker plasmonic response than 200 °C, whereas 250 °C produced significantly lower performance. As higher temperatures are associated with an increased risk of substrate degradation and the enhancement was the highest of the tested temperatures, 200 °C applied for 1 h was selected for the dewetting process. It balances well the risk of PDMS degradation and oxide formation as well as plasmonic performance.

**Figure 1:**
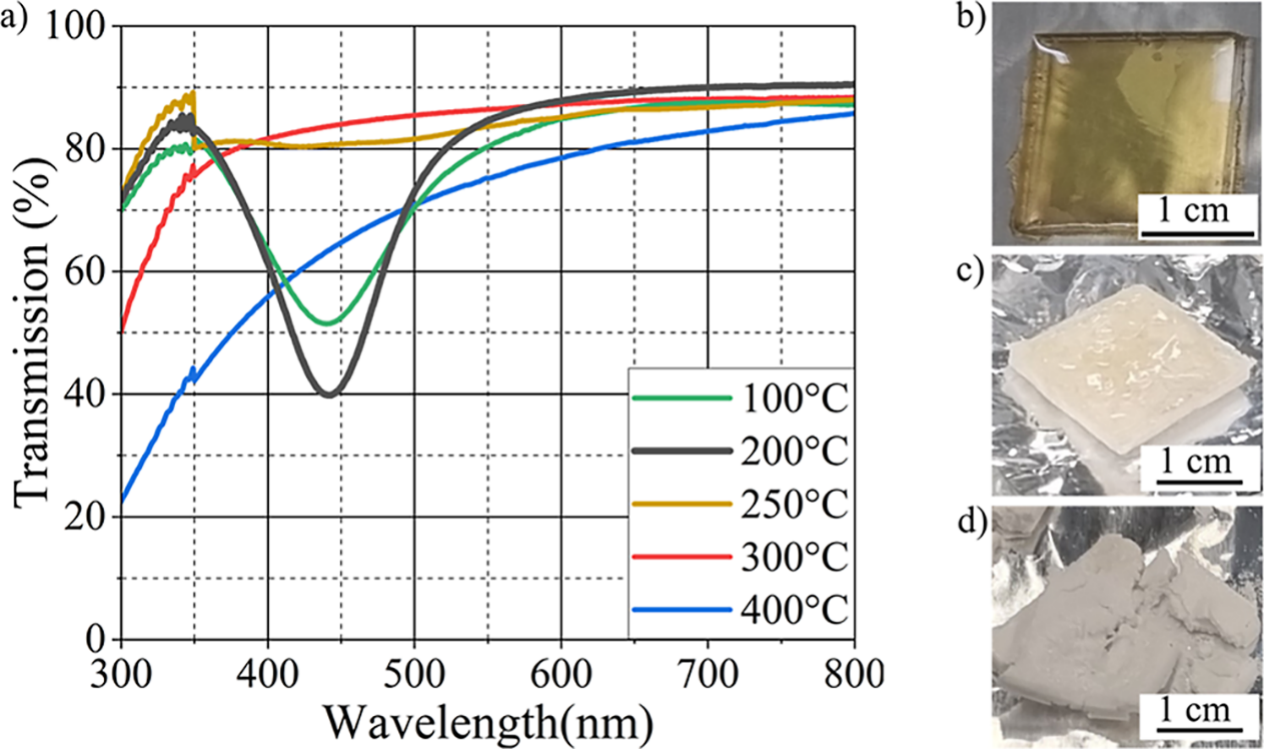
(a) Transmission spectra of dewetted samples with an initial silver layer thickness of 3 nm for different temperatures ranging from 100 to 400 °C (1 h annealing duration), (b) exemplary image of a sample before the annealing, (c) same sample after 500 °C for 1 h, and (d) complete loss of the flexibility and structural integrity of the PDMS after applying soft pressure with a tweezer.

Dewetting in air leads to partial oxidation of the formed silver nanoparticle layer during the process. This effect was investigated before on glass samples, where higher temperatures led to increased oxidation while still producing controllable plasmonic responses.^53^ Therefore, our low-temperature regime should minimize oxide capping layer formation compared with standard high-temperature dewetting processes.

The repeatability of the process was additionally investigated on five samples in separate process flows with an exemplary initial thickness of 9 nm. For this preliminary experiment, we also used the same process parameters, meaning 200 °C annealing for 1 h. The samples showed similar trends in the resulting transmission spectra like the ones presented in Figure 3 with slight deviations. The mean characteristics of these samples as well as their standard deviation can be seen in Table 1. A slight shift in peak resonance wavelength was observed, resulting in a mean peak wavelength of 472.0 ± 2.5 nm. Minor variations in the magnitude of the dip and the FWHM were also detected, leading to slightly altered *Q* factors. These deviations are a combination of deviations in the process parameters and the measurement resolution of the used systems. Therefore, the samples that are presented together in transmission spectra were processed together to minimize the process-related influences and to make a comparison as precise as possible.

**Table 1:** Investigation of the Repeatability of the Presented Process on Dewetted Samples with an Initial Thickness of 9 nm

initial thickness [nm]	λ_0_ [nm]	FWHM[nm]	magnitude [%]	*Q* factor
9	472.0 ± 2.1	209.0 ± 10.7	62.88 ± 3.16	2.26 ± 0.13

### 3.2 Fabrication Process of Soft Plasmonic Masks

The fabrication of the soft plasmonic masks was carried out in five steps (Figure 2). To investigate the influence of surface structuring, two types of masters were used: planar, unstructured glass samples (Figure 2I) and an in-house-fabricated microstructured silicon master for structured samples (Figure 2II). Prior to use, both types of masters underwent a cleaning process consisting of 5 min acetone treatment, 5 min treatment in isopropanol (ISO), rinsing in deionized water, and finally drying with gaseous dry nitrogen using a nitrogen gun (Figure 2a). PDMS was subsequently poured into 3D-printed molds that were attached to the master. These molds were used to ensure uniform thickness and dimensions across all fabricated samples (Figure 2b).

**Figure 2:**
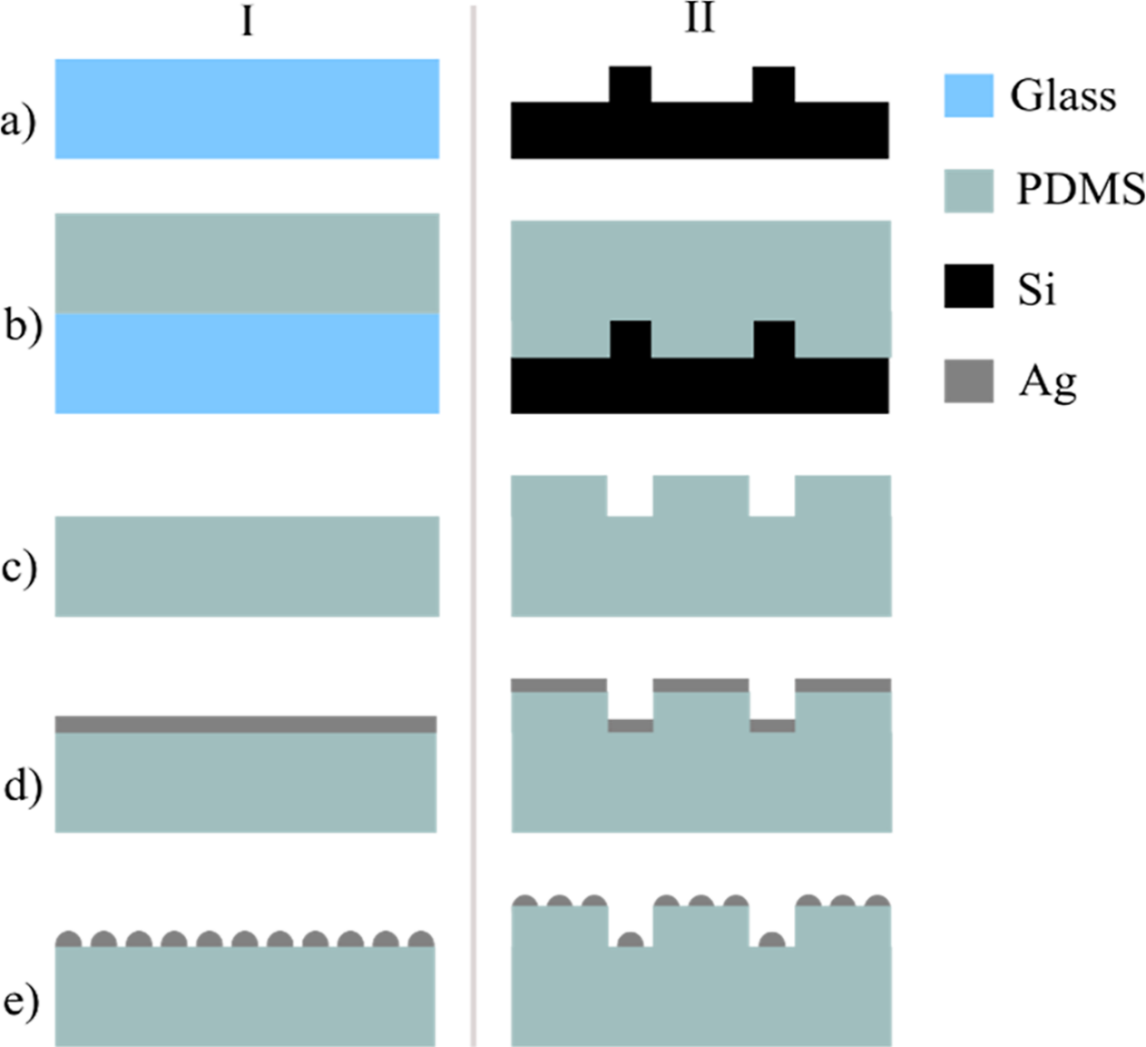
Flowchart of the fabrication of the soft plasmonic masks: (a) preparation of the masters, (b) PDMS curing, (c) PDMS demolding, (d) metallization, and (e) dewetting. I—planar glass master and II—structured silicon master.

The PDMS samples were carefully demolded from the masters (Figure 2c) after curing, thereby transferring a negative replica of the master structure onto the PDMS surface. For the glass samples, this resulted in a flat unstructured surface with the roughness defined by the master, whereas the structured samples reproduced the grid pattern as a negative replica, with square areas measuring 30 μm side length and 4.2 μm height while maintaining the original pitch of 40 μm.

To metallize the thin silver layers, electron-beam evaporation was performed at a controlled evaporation rate of 0.04–0.05 nm/s (Figure 2d). The relatively low evaporation rate was selected to ensure precise control over the created thin-film thickness, to minimize surface roughness, and to reduce substrate surface stress.^54^ The side being previously in contact with the master was coated, and the film thickness was continuously monitored by using an internal QCM integrated into the deposition tool. Precise control over the deposited thickness is an important parameter, as rather small deviations can lead to drastic changes in the resulting performance of the fabricated sample. Since PDMS substrates are sensitive to vacuum exposure and metal evaporation, careful handling is required to minimize tensile stress, which can induce morphological changes and surface wrinkling.^55^ For applications involving both the masks and the dewetting process, minimal surface roughness is particularly essential, as increased roughness reduces the plasmonic enhancement, as demonstrated previously in a study on metallized PDMS samples used in SERS applications.^56^

For the annealing process, a hot plate operated under ambient conditions was used and set to the target temperature, 200 °C, described in more detail in Section 3.1. The PDMS side, meaning the nonmetallized side of the soft mask, was placed in contact with the preheated hot plate. After 1 h, the samples were carefully removed and cooled down (Figure 2e).

### 3.3 Evaluation of Planar PDMS Samples

First, the samples with planar, meaning unstructured surfaces that were fabricated using glass masters, are discussed in the following section. All samples presented in this section were produced according to the process flow shown in Figure 2 (left, I).

The transmission measurements of the fabricated soft masks with silver film thicknesses of 1 to 9 nm are shown in Figure 3a. The black line shows the transmission spectrum of a nonmetalized but dewetted PDMS sample, serving as a reference to confirm that the observed optical changes do not arise from deformations in the PDMS layer. In general, as the initial film thickness increases, less light is transmitted through the sample, and the overall transmission decreases. All studied samples exhibited a characteristic plasmonic dip, indicated by the plasmonic band in Figure 3a that redshifts slightly with increasing initial thickness. The key parameters resonance wavelength, full width half-maximum (FWHM), magnitude of the plasmonic dip, and *Q* factor of each measurement are furthermore summarized in Table 2 for comparison purposes.

**Figure 3:**
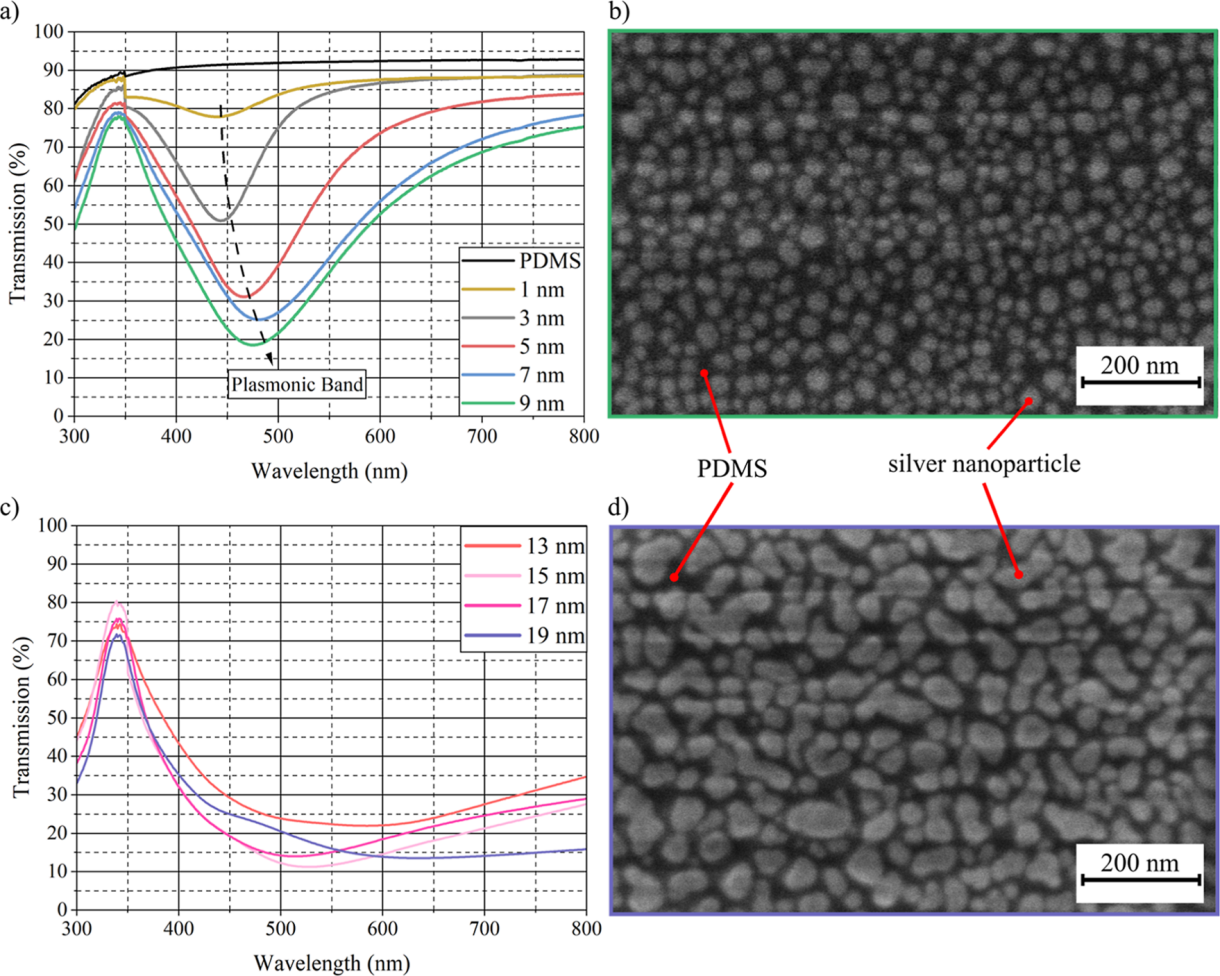
Transmission spectra of dewetted samples with silver layers ranging from (a) 1 to 9 nm and (c) 13 nm to 19 nm; SEM micrograph of a sample with initial silver layer thicknesses of (b) 9 nm and (d) 19 nm after dewetting. Silver nanoparticles appear brighter, while dark areas represent the PDMS substrate.

**Table 2:** Comparison of Parameters Obtained from the Transmission Measurements for Samples with Initial Ag Layer Thicknesses Ranging from 1 to 9 nm

initial thickness [nm]	λ_0_ [nm]	FWHM [nm]	magnitude [%]	*Q* factor
1	440	143	10.60	3.08
3	444	**97**	37.89	**4.58**
5	446	151	48.77	2.95
7	481	184	53.88	2.61
9	475	190	**59.44**	2.50

A pronounced dip at the resonance wavelength λ_0_ indicates a strong plasmonic effect. Another important parameter is the FWHM, where smaller values correspond to higher sensitivity, which is of relevance for sensing applications.

Additionally, the *Q* factor, described previously in Section 2.2, was calculated to quantify resonance sharpness and compare performance across fabricated samples, where higher values indicate superior narrowness and quality.^40,41^

The results show that increasing the initial silver thin-film thickness affects several key optical properties of the soft plasmonic masks. Samples with greater layer thickness exhibit a redshift of the resonance wavelength. Since the intended application targets shorter wavelengths in the near-UV range, thinner films are therefore advantageous. Thicker layers also lead to a spectral broadening of the plasmonic band, reflected by larger FWHM values, reduced sensitivity, and consequently a lower *Q* factor. In addition to this broadening, the resonance minima become less sharp, which further diminishes sensitivity and correlates with the larger particle sizes originating from thicker initial layers. In contrast, thicker layers exhibit a greater dip, reflecting an enhanced resonance strength.

The redshift is quantitatively demonstrated by the resonance wavelength shifting from 440 to 475 nm as the initial layer thickness increases. Regarding the FWHM, the 3 nm sample shows the lowest value of 97 nm, indicating the highest sensitivity, whereas the 9 nm sample shows the largest value of 190 nm, corresponding to lower sensitivity. The expected opposite trend is observed for the resulting *Q* factor values which range from 2.50 for the 9 nm sample up to the highest value of 4.58 for the 3 nm sample. Regarding the dip magnitude, the thinnest investigated layer of 1 nm exhibits the lowest value of 10.60%, while the highest value is found for the 9 nm sample at 59.44%. In general, all these samples are suitable for the intended application as soft plasmonic masks, but the most promising ones are the 3 and 9 nm layers. The 3 nm sample achieves the highest *Q* factor of 4.58, while the 9 nm sample shows the deepest dip of 59.44%.

The higher dip magnitude indicates a stronger plasmonic enhancement at the resonance wavelength. Therefore, the sample with an initial silver thin-film thickness of 9 nm is better suited for plasmonic lithography or SERS sensing, where high-field enhancement at a well-defined wavelength is more important than resonance broadening.^14^ In contrast, the sample initially covered by a 3 nm-thick silver film exhibits a higher *Q* factor due to its narrower FWHM. This makes it more suitable for sensing applications that require a high spectral precision. The narrower resonance allows, for example, for more accurate detection of local refractive index changes with a reduced noise. Thereby, the sensing resolution compared to the other thicknesses investigated is improved.^57^

The realistically achievable *Q* factor depends on several factors, such as the physical working principle, the metal used, and the employed fabrication technique. For more complex systems, high *Q* factors were reported, often in the 200–300 range^41,58^ and sometimes even exceeding 2000.^59^ These reported values are usually linked to additional effects like surface lattice resonance and the use of noble metals like Au that are not prone to oxidation effects.

More comparable plasmonic systems are therefore based on LSPR that usually show lower *Q* factors of >10 ^16^ due to higher losses and damping effects.^60^ Most research using dewetting on Ag thin layers focuses on applications such as SERS rather than on *Q* factor-optimized systems. As a result, the *Q* factors are not always reported directly. Nevertheless, they can be estimated from the presented spectra to be in a range comparable to the values we achieved.^34,50^

The transmission measurements of the fabricated soft masks with an Ag film thickness of 13 to 19 nm are shown in Figure 3c. The curves exhibit either a very weak and broad plasmonic dip or no discernible dips at all. For these samples, the characteristic parameters could not be calculated reliably. Treating the curve as a broad dip would position the baseline outside the measurement range and prevent accurate quantification. Even if the curves are interpreted as a broad dip, the *Q*-factors would be very low and the plasmonic performance poor. Consequently, samples with initial silver layer thicknesses between 11 and 19 nm do not appear suitable for the fabrication of soft plasmonic masks based on the presented dewetting process.

While thinner film thickness yielded a surface with small nanoparticles of varying sizes and relatively circular shapes, a thicker film exhibited comparatively larger particles with greater variability in size and shape after dewetting. Representative SEM images of nanostructured samples with initial silver film thicknesses of 9 and 19 nm are shown in Figure 3b,d, respectively.

Previous studies showed that size and shape are critical parameters for nanostructured thin films in plasmonic applications: larger particles lead to a redshift, while the smaller ones induce a blueshift. Additionally, deviations from circular symmetry also result in a redshift and further reduce the strength of the plasmonic resonance. SEM images were used to evaluate therefore the mean particle size, shape, and size distribution of the nanoparticles (Figure 4). However, geometric analysis by SEM becomes more challenging for thinner metal layers because of increased charging, which slightly blurs the particle edges. Nevertheless, a clear trend can still be observed by using this method. The presented diameter values are rounded to full nanometer values to be in alignment with the lateral resolution of the used SEM. The circularity value quantifies how closely a structure approximates a perfect circle with 1 corresponding to an ideal circular shape. The determined distributions are presented as histograms in Figure 4a–c for a sample with initially 3, 9, and 19 nm silver film thickness, respectively. The 3 nm sample was chosen for its highest *Q* factor among all tested samples. The 9 nm sample represents the thickest film that still shows a clear plasmonic effect, characterized by the most pronounced spectral dip. In contrast, the 19 nm sample, included as the thickest film investigated, no longer exhibits a plasmonic response. The comparatively small particle sizes may be related to the initial morphology of the Ag layer on PDMS, which is likely not fully continuous prior to annealing. In addition, local substrate topography may influence surface diffusion and the characteristic dewetting length scale during solid-state dewetting.^61^

**Figure 4:**
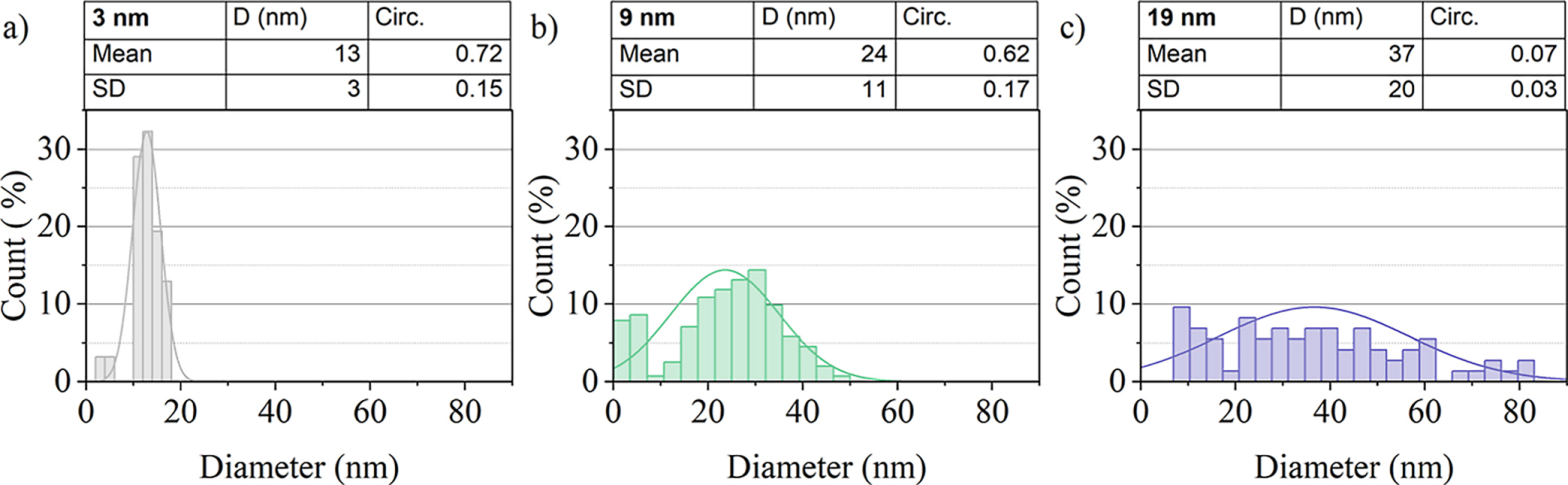
Diameter distribution histogram, mean particle sizes D, standard deviation SD, and circularity (Circ.) of silver nanoparticles observed after the dewetting process based on an initial silver film thickness of (a) 3 nm, (b) 9 nm, and (c) 19 nm. SD denotes the standard deviation.

The mean particle diameter for the 3 nm sample is 13 nm, with most particle diameters in the size range of 10 to 18 nm (Figure 4a). These nanoparticles also exhibit a relatively high circularity of 0.72 and are fairly uniform in size. High uniformity and symmetry are advantageous for the plasmonic performance because they result in narrower and more pronounced resonance dips. This is also reflected in the measured and calculated results, where the 3 nm initial thickness sample shows the highest achieved *Q*-factor in this study. As expected, the particle sizes for the 9 nm sample range from 15 to 40 nm with a mean diameter of 24 nm (Figure 4b). Their mean circularity is 0.62, indicating a trend toward more irregular shapes. These characteristics, namely, the moderate uniformity and circularity, are reflected in the observed larger plasmonic dip, redshifting of the resonance wavelength, and increased FWHM values (cf. Figure 3a).

The particle sizes of the thickest investigated silver layer of 19 nm range widely from about 10 nm up to 80 nm, with a mean diameter of 37 nm (Figure 4c). The shapes deviate significantly from an ideal circular shape, as indicated by the comparatively low mean circularity value of 0.07. The broad distribution of particle sizes and low symmetry lead to the superposition of multiple plasmonic resonances from nanoparticles of different sizes, resulting in a broadening, a redshift of the resonance, and a vanishing of a distinct dip (cf. Figure 3c).

Another important parameter is the interparticle gap, which describes the distance between individual nanoparticles. As the distance increases while their size remains constant, a blueshift of the observed spectra and a reduction in plasmonic enhancement are expected. With larger interparticle gaps, the plasmonic response approaches that of isolated nanoparticles, as the electromagnetic coupling weakens.^62–64^

To evaluate the samples in this regard, the interparticle gaps were measured and are summarized in Table 3. All investigated samples exhibit relatively large standard deviations compared with their respective mean values. This behavior arises from the nonuniform spatial arrangement of the nanoparticles, which is observed across all samples.

**Table 3:** Results of the Measured Mean Interparticle Gap of Samples with Initial Film Thicknesses of 3 nm, 9 nm, and 19 nm and Their Standard Deviation (SD)

initial thickness [nm]	mean interparticle gap [nm]	SD [nm]
**3**	5	3
**9**	5	3
**19**	8	5

When the initial thickness increases from 3 to 9 nm, no significant difference in the interparticle gap is observed. Both samples exhibit a mean interparticle gap of 5 nm with a standard deviation of 3 nm. However, this does not necessarily indicate that the true interparticle distances are identical. This similarity likely stems from the limited resolution of the SEM images, which is particularly true for the 3 nm-thick sample, where image noise represents a significant influencing factor.

A comparison between the 9 and 19 nm samples reveals that a larger initial film thickness leads to an increased mean interparticle distance, in this case of 8 nm. This increase is accompanied by a decrease in the plasmonic response, which agrees with the optical measurements. Furthermore, the reduced uniformity previously observed in the particle diameter distribution is also reflected in interparticle gap analysis. The sample with a 19 nm-thick initial silver layer exhibits a higher standard deviation, indicating a broader distribution of measured distances.

In summary, our experiments confirm the expected impact of the mean shape and size of nanoparticles as well as of the interparticle distance on the plasmonic performance. Structures with overall uniform nanoparticle sizes and more symmetric shapes exhibit stronger and sharper resonances.

### 3.4 Long-Term Stability of Planar PDMS Samples

The long-term stability is a critical factor for potential real-world applications, encompassing both the adhesion of the metal structures to PDMS and their structural and optical integrity. Due to its flexible, elastomeric nature, PDMS can pose challenges for the stability and adhesion of deposited metal layers.^55^ In our experiments, all handling and analysis steps were therefore carefully monitored in this regard. However, no adhesion issues were observed, which demonstrated the overall robustness of the fabricated samples. The optical performance integrity was representatively examined for two of the most promising soft plasmonic masks, namely, samples with 3 and 9 nm initial silver film thickness. The optical transmission properties were investigated after fabrication and the initial characterization as well as after prolonged storage under cleanroom conditions for six months. Both samples showed a reduced plasmonic performance, as shown in Table 4. This is primarily attributed to oxidation of the silver layer and maybe to potential changes in particle aggregation or morphology.^65,66^

**Table 4:** Results of the Long-Term Stability Experiments Showing a Parameter Comparison of Samples with Initial Silver Layer Thicknesses of 3 and 9 nm with Δ Being Defined as the Difference between the Initial Values and the Values Observed after Six-Month Storage under Cleanroom Conditions

initial thickness [nm]	Δ λ_0_ [nm]	Δ FWHM [nm]	Δ magnitude [%]	Δ *Q* factor
3	1	–20	13.37	0.79
9	–4	–66	1.26	0.63

The degradation was more pronounced for the 3 nm samples, which is consistent with findings that silver nanoparticles smaller than 5 nm are more vulnerable to environmental effects^67^ and quantum size effects can further amplify this degradation. In result, an increased damping and a 13.37% reduction in the dip magnitude were observed for the 3 nm sample. However, the resonance wavelength remained almost constant, whereas the plasmonic peak broadened, with the FWHM value increasing by 20 nm. Consequently, the *Q* factor decreased as well by approximately 0.79 to a value of 3.78, which remains still higher than the *Q* factors observed for other investigated samples.

In comparison, the 9 nm sample was mainly affected by surface oxidation, showing only a 1.26% decrease in dip magnitude and only a slight redshift of 4 nm for the resonance wavelength. However, the plasmonic dip broadened significantly, which is indicated by an increase in FWHM of 66 nm. This change leads to a *Q* factor decrease of 0.63, resulting in a *Q* factor of 1.87 for the 9 nm sample after six months of storage. Since the initial *Q* factor of the 9 nm sample was already lower than the value observed for the 3 nm sample, a decrease of similar magnitude corresponds to a larger relative drop in performance. The observations indicate overall that the plasmonic soft masks can still be effectively used after an extended storage, even though their performance declines significantly. Thinner silver layers, such as the 3 nm film, provide higher initial *Q* factors but experience larger decreases in dip magnitude while still maintaining reasonable *Q* factors. Thicker layers, like those near 9 nm, show significant dip broadening and slight redshifts, which contribute to more distinct *Q* factor reductions. Some studies suggest that applying a very thin protective coating can help prevent oxidation, thereby mitigating *Q* factor degradation and preserving the optical properties.^68–71^ The choice of protective material must also be carefully considered to maintain the initial performance and the position of the plasmonic resonance peak.

However, our results do not follow the observed trends reported in ref 70 where plasmonic activity without a protective coating degraded rapidly: by 20% after 20 days and approximately 80% after 175 days (almost six months). In that study, the FWHM of the normalized absorbance was used to characterize plasmonic activity. In contrast, our observed changes in FWHM after 180 days (six months) were 20.62% for the 3 nm sample and 37.74% for the 9 nm sample.

This higher stability of our samples could be attributed to the formation of an oxide layer under our experimental conditions, which develops during the initial dewetting step in ambient conditions and slows further oxidation of the silver nanoparticles.^72^ It thereby reduces the degradation of the plasmonic effect. Nevertheless, the strategy proposed in ref 72 could lead to further improvements in long-term stability.

### 3.5 Evaluation of Structured PDMS

For applications of plasmonic masks in lithographic or sensing applications, it is frequently necessary to adapt the size of the active region. To demonstrate that localization of the plasmonic effect is achievable, we used the previously described process flow illustrated in Figure 2 (right, II) with an initial silver layer of 3 nm thickness to fabricate structured soft plasmonic masks. This initial silver layer thickness was chosen on the basis of the promising results observed for the planar samples, as discussed above. Its resonance wavelength of 440 nm lies within the targeted near-UV region of the optical spectrum and could, for example, be used in lithographic applications with a broadband UV photoresist. Additionally, samples with this initial layer thickness exhibited the highest *Q*-factors among those analyzed, while retaining a pronounced plasmonic dip, making them the most promising candidates for sensing applications.

In contrast to the previous samples, these plasmonic masks were fabricated using structured masters, as described in Section 2.1. An additional evaluation step is required to confirm the accurate transfer of the grid structure into the PDSM and the successful metallization prior to dewetting. This validation is crucial for assessing optical performance and results. Deviations can help to identify specific fabrication steps contributing to discrepancies. A LSM image of the structured sample with a 3 nm silver layer metallization is shown in Figure 5. Figure 5a presents a top view of the structure, demonstrating the accurate and successful transfer of the pattern, across a larger area. A representative area of this structure, as indicated by an orange square in Figure 5a, is shown in a 3D view of the LSM image in Figure 5b. The red line in both images indicates the location of the extracted representative line profile shown in Figure 5c. The shown profile confirms the accurate transfer of the master, as indicated by a matching height, width, and pitch of 4.2 μm, 30 μm, and 40 μm, respectively, for both the master and PDMS structures.

**Figure 5:**
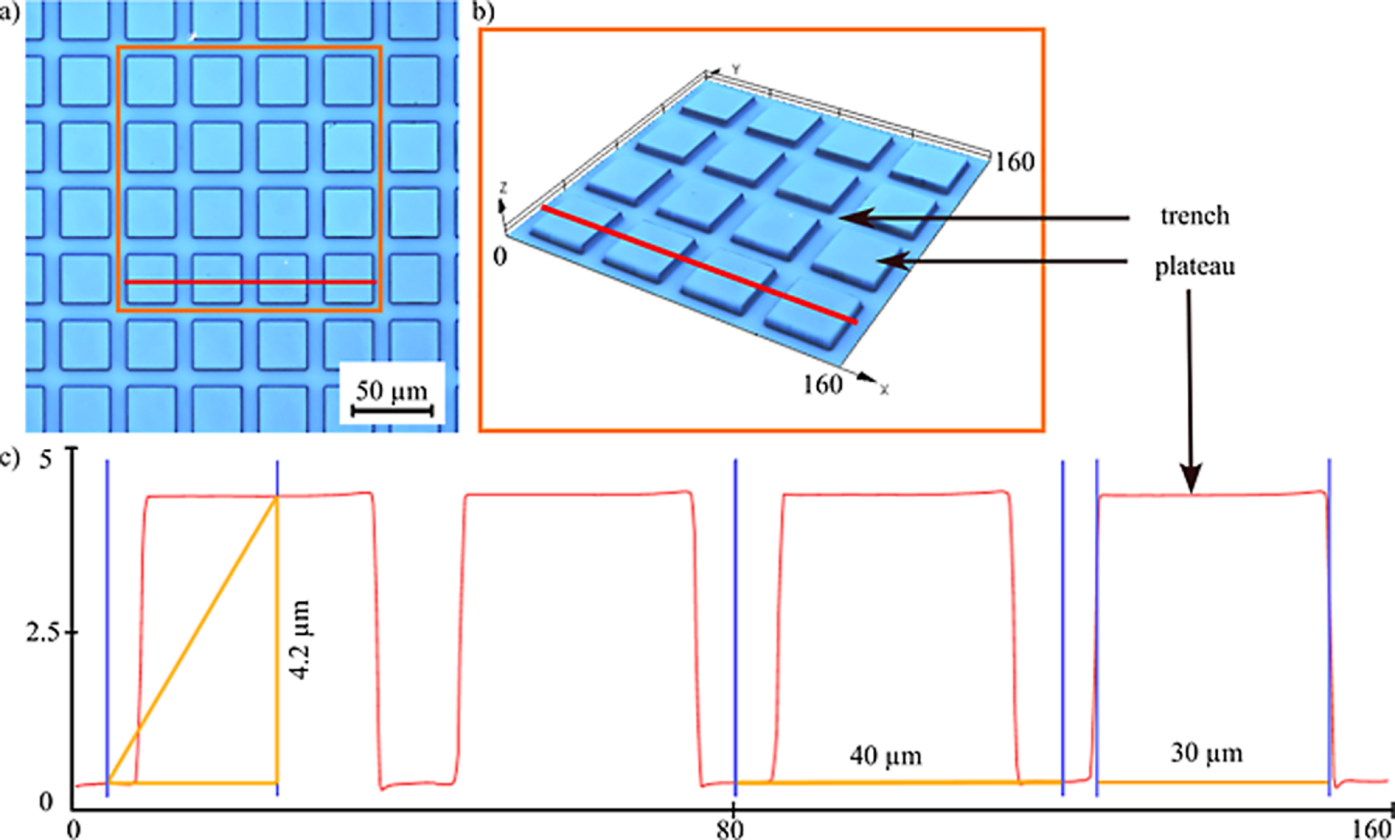
Structured sample with an initial 3 nm Ag layer; (a) LSM top view of the structured surface; (b) 3D view of the LSM scan; and (c) line profile of the sample for the gray line depicted in (b) with the measured height of the trenches of 4.2 μm, the pitch of the structure of 40 μm, and the width of the plateaus of 30 μm matching the silicon master.

After transfer of the grid structures from the master, silver metallization, and dewetting, the optical performance of the soft plasmonic mask was evaluated and compared (Figure 6a). A SEM image of a representative plateau area on a structured PDMS after silver layer dewetting is additionally shown and confirms the successful formation of the intended silver nanoparticles (Figure 6b). To confirm that the observed optical effects are not attributable to deformations of the intrinsic material structure, a nonmetalized but structured PDMS sample was used as control (black line in Figure 6a). This reference sample was treated identically to the dewetted metalized samples but did not exhibit a significant change in optical performance. The structured sample metallized with an initial silver layer thickness of 3 nm already exhibits a plasmonic dip prior to the dewetting process (dashed line in Figure 6a).

**Figure 6:**
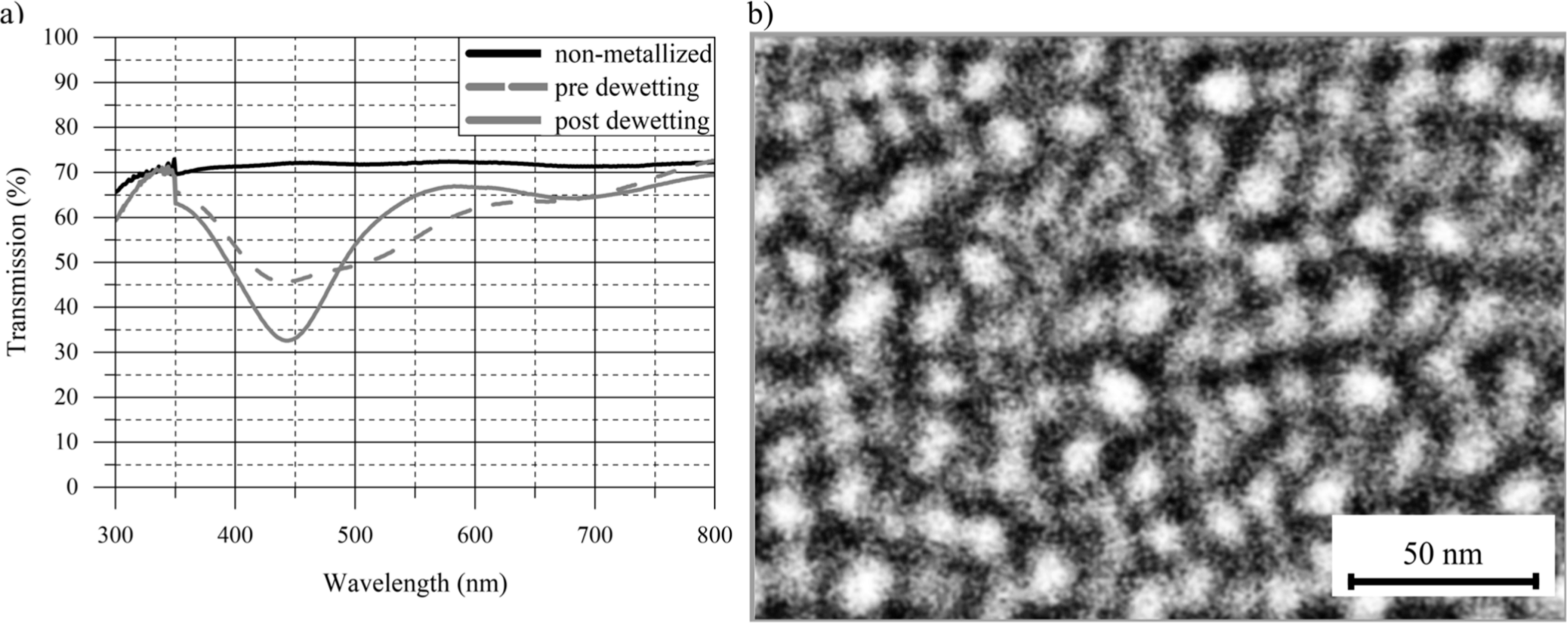
(a) Transmission spectra of the nonmetallized PDMS, the sample before dewetting and after it; (b) SEM showing a structured surface after silver layer dewetting. Charging effects slightly reduce the image quality. Silver nanoparticles appear brighter in the image, while the dark areas represent the PDMS substrate.

This indicates that the silver film was not fully continuous prior to the dewetting process, which is expected given the low nominal film thickness of 3 nm. Previous studies showed that very thin silver layers tend to be either porous or islandlike and therefore do not form a continuous film. As a result, such films already resemble a nanostructured surface, which gives rise to a weak plasmonic resonance response.^73,74^ The optical performance of the dewetted structured soft plasmonic mask shows in accordance an even more pronounced plasmonic dip (solid gray line in Figure 6a). The occurrence of the observed double dip in the spectra at about 442 and 700 nm likely originates from the structuring of the surfaces. This so-called spectral splitting can be attributed to the presence of nanoparticles within the trenches, which introduce a displacement of the localized plasmon modes along the *z* direction and results in a double-dip resonance pattern. A similar behavior has been reported for plasmonic nanostructure arrays that integrate both metallic and dielectric components.^57,75^ By analogy, the observed double dip can be interpreted as the formation of two hybrid modes associated with particles on the flat regions and inside the trenches, respectively, whose different coupling strengths and radiative losses result in the experimentally observed spectral splitting.

The key parameters were again extracted from optical transmission measurements and enabled a comparison of structured samples to those prior to dewetting and the planar samples, which are quantified in Table 5. Additionally, the results for the planar sample with an initial silver thickness of 3 nm, described in detail in Section 3.3 are added for ease of comparing the planar to the structured results. Comparison of the spectra prior to and after dewetting reveals several changes. The dip magnitude increases by about 10% from 38.54% to 27.06%. At the same time, the FWHM decreases significantly from 191 to 106 nm, leading to a narrower resonance band. Consequently, the *Q* factor increases from 2.31 to 4.18, which is a direct result of the increased dip magnitude and the narrower FWHM. The resonance wavelength, however, remains almost constant, which can be also observed for the second dip at around 700 nm, which is not affected by the dewetting process.

**Table 5:** Comparison of Parameters Obtained from the Transmission Measurements for the Structured Samples with an Initial Silver Layer Thickness of 3 nm for Post- and Pre dewetting and a Dewetted but Planar Reference Sample

initial thickness 3 nm	λ_0_ [nm]	FWHM [nm]	magnitude [%]	*Q* factor
predewetting-structured	442	191	27.06	2.31
structured	443	106	38.54	4.18
planar	444	97	37.89	4.58

Similarly, a comparison of the spectra of the dewetted structured sample with the planar sample shows that the resonance wavelength remains unchanged. The dip magnitude is comparable, while the FWHM is slightly broader by 9 nm, resulting in a correspondingly lower *Q* factor by approximately 0.4. These results confirm that the dewetting process is robust on structured PDMS samples, highlighting their versatility for various applications.

Optimization and further exploration of the design and geometry of the structured PDMS substrate could open avenues for more advanced research. Adopting soft, nanostructured surfaces analogous to templated dewetting approaches on rigid substrates^30^ may improve nanoparticle ordering and consequently enhance performance.

Additionally, optimization trench height could strengthen coupling, with shallower trenches promoting higher enhancement and coupling of the particles displaced along the *z*-axis,^76^ potentially enabling the fabrication of samples with higher *Q* factors.

## 4 Conclusion

In conclusion, this work demonstrates a simple yet versatile method for fabricating tunable soft plasmonic masks on flexible PDMS substrates via solid-state dewetting of thin silver films. The resulting masks exhibit well-defined plasmonic resonances that can be precisely tuned by varying the initial film thickness. The most promising samples were fabricated from a 3 nm-thick silver layer, which achieved the highest *Q*-factor (4.58), whereas an initially 9 nm-thick silver layer produced the strongest transmission dip (59.44%). These performance metrics are slightly lower in comparison to those of solid-state dewetting on rigid substrates, confirming the viability of soft plasmonic platforms despite their relative simplicity.

Long-term stability tests conducted after six months revealed that the optical properties remained sufficiently robust, with only minor degradation due to oxidation. The compatibility with structured substrates enables spatially localized plasmonic effects, offering, for instance, control for nanolithographic patterning and sensing applications such as SERS and higher resolution for refractive-index sensing. Future improvements through template-assisted dewetting, smaller design features, or protective coatings should further enhance spectral performance and stability, broadening the applicability of these flexible plasmonic masks in advanced nanophotonics and sensing technologies.
